# Experimental Infection of Plants with an Herbivore-Associated Bacterial Endosymbiont Influences Herbivore Host Selection Behavior

**DOI:** 10.1371/journal.pone.0049330

**Published:** 2012-11-14

**Authors:** Thomas Seth Davis, David R. Horton, Joseph E. Munyaneza, Peter J. Landolt

**Affiliations:** USDA-ARS, Yakima Agricultural Research Laboratory, Wapato, Washington, United States of America; Max Planck Institute for Chemical Ecology, Germany

## Abstract

Although bacterial endosymbioses are common among phloeophagous herbivores, little is known regarding the effects of symbionts on herbivore host selection and population dynamics. We tested the hypothesis that plant selection and reproductive performance by a phloem-feeding herbivore (potato psyllid, *Bactericera cockerelli*) is mediated by infection of plants with a bacterial endosymbiont. We controlled for the effects of herbivory and endosymbiont infection by exposing potato plants (*Solanum tuberosum*) to psyllids infected with “*Candidatus* Liberibacter solanacearum” or to uninfected psyllids. We used these treatments as a basis to experimentally test plant volatile emissions, herbivore settling and oviposition preferences, and herbivore population growth. Three important findings emerged: (1) plant volatile profiles differed with respect to both herbivory and herbivory plus endosymbiont infection when compared to undamaged control plants; (2) herbivores initially settled on plants exposed to endosymbiont-infected psyllids but later defected and oviposited primarily on plants exposed only to uninfected psyllids; and (3) plant infection status had little effect on herbivore reproduction, though plant flowering was associated with a 39% reduction in herbivore density on average. Our experiments support the hypothesis that plant infection with endosymbionts alters plant volatile profiles, and infected plants initially recruited herbivores but later repelled them. Also, our findings suggest that the endosymbiont may not place negative selection pressure on its host herbivore in this system, but plant flowering phenology appears correlated with psyllid population performance.

## Introduction

Endosymbiotic associations are important biological systems for testing ecological concepts [1]. In particular, interactions between plants, herbivorous arthropods, and their microbial endosymbionts have received much research attention [2], [3], and an impressive array of endosymbiotic relationships are common among certain herbivore taxa (e.g., Aleyrodidae, Aphididae, Cicadellidae, Glossinidae, Psyllidae, Psuedococcidae, Scolytinae, Tenebrionidae, etc.) [4], [5]. These symbioses have a range of effects on herbivore populations, and symbionts can be significant in providing nutrition [2], [6], [7], determining sex ratios [8]–[10], mediating arthropod interactions with predators and pathogens [11], [12], detoxifying plant secondary compounds [13], chemical signaling [14], and more.

Several recent works point to an emerging trend in plant-herbivore-endosymbiont ecology: in some cases, plants that have been colonized by endosymbionts reduce herbivore fitness [15]–[17], theoretically placing negative selection on the association. How, then, can associations with antagonistic endosymbionts persist over evolutionary time? The current predominant hypothesis is that plants infected with endosymbionts exhibit altered volatile chemistry that increases the attractiveness (via olfaction) of plants to herbivores [15]–[18]. In one example, this effect was correlated with the induction of a single volatile compound [16], though in other studies herbivore behaviors were correlated with the total abundance of plant volatiles emitted [15]. Yet, herbivores apparently defect from host plants that are initially attractive but are also ultimately associated with a negative fitness cost [16]. Collectively, these studies suggest that migration from one host to another during the life of an individual herbivore is essential for the maintenance of negative symbiotic associations. However, nearly all previous studies rely on tissue grafts for the transmission of pathogenic endosymbionts to plants, which could obscure potential interactions between the effects of herbivory and the effects of herbivory plus endosymbiont infection on plant signals and subsequent herbivore behaviors.

Here, we report experiments designed to link plant infection with a bacterial endosymbiont to herbivore behaviors and population dynamics. Our experiments investigate interactions between the potato/tomato psyllid (*Bactericera cockerelli* Sulc.), a fastidious endosymbiotic α-proteobacterium (“*Candidatus* Liberibacter solanacearum”; LB), and host potato plants (*Solanum tuberosum* L.). *Bactericera cockerelli* are inconspicuous, polyphagous herbivores that oviposit and develop on a wide range of host plants in the Solanaceae family [19]. Consequently, *B. cockerelli* is generally regarded as a pest species that cyclically causes damage to commercial agricultural operations. Although the symbiosis between *B. cockerelli* and “*Ca*. L. solanacearum” is only recently described [20], [21], it has raised tremendous concern among growers: plants that become infected with *Ca*. L. solanacearum rapidly wilt and die, tuber yield is greatly reduced, and symptomatic tubers are unmarketable [20]. Despite these concerns, the general importance of endosymbionts in *B. cockerelli* behaviors and population performance remain unknown (but see [22]).

We addressed three questions: (1) Are plant volatile profiles altered by infection with endosymbionts? (2); Is host selection by herbivores (*B. cockerelli*) related to plant infection status?; and (3) Do infected plants have consequences for herbivore population growth? We separated the effects of herbivory on host plants from endosymbiont infection by exposing plants to psyllids infected with “*Ca*. L. solanacearum” (LB+) or uninfected psyllids (LB−). The abundance and composition of volatile emissions from plants differed modestly with respect to both herbivory and endosymbiont infection. In cage release experiments, psyllids initially settled on infected plants but subsequently defected to uninfected plants. However, we found no evidence that plants infected with the pathogenic endosymbiont diminished psyllid population growth or egg production, but flowering by potato plants was correlated with a decrease in psyllid performance.

## Materials and Methods

### *B. cockerelli* colonies

Colonies of *B. cockerelli* are maintained at the USDA-ARS Yakima Agricultural Research Laboratory (Wapato, WA, USA) by J. Munyaneza and D. Horton. Colonies are maintained in multiple 60 cm×60 cm×60 cm mesh tents (BugDorm 2120F, Lot no. BD2120, BioQuip, Rancho Dominguez, CA) in climate-controlled rooms (24°C, 40% R.H., 16∶8 L∶D). Colonies that are positive (infected) for “*Ca*. Liberibacter solanacearum” are kept in separate rooms to prevent cross-contamination. The infection statuses of uninfected and infected psyllid colonies are confirmed at regular intervals by amplification of 16S rDNA using the polymerase chain reaction (PCR) and the primer pair OA2/OI2 (described in [23], [24]). Multiple infected and uninfected colonies are simultaneously maintained, and colonies are occasionally supplemented with psyllids from natural populations. Colonies are provided with potted *S. tuberosum* (var Atlantic) ad libitum, and psyllid colonies were initially propagated from natural populations collected in the vicinity of Moxee, WA, USA (46.57°N 120.41°W, 400 m a.s.l.) and Weslaco, TX, USA (26.16°N 97.98°W, 20 m a.s.l.).

### Plant growth conditions and experimental treatments

We established all *S. tuberosum* plants used in our experiments from mini-tubers (var Atlantic, CSS Potato Farms LLC, Colorado City, CO) by growing tubers in a compressed bale mix (McConkey Co., SUNSM4, Sumner, WA) in a greenhouse (27°C, 40% R.H., 16∶8 L∶D). After 21 d of growth under these conditions, we experimentally exposed plants to psyllids: plants were challenged with LB− psyllids (uninfected psyllid treatment) or LB+ psyllids (infected psyllid treatment). We caged each plant individually with organdy mesh (0.5 mm×0.5 mm) supported by wire. We collected psyllids by aspiration from colonies at random into 15 ml polyethylene scintillation vials, and we released a total of 10 psyllids onto each individually caged plant by placing open vials under mesh caging. A subset of plants was caged but not further treated (undamaged control). After 24 h, we carefully removed cages and extracted psyllids from plants by aspiration.

### Do challenged plants exhibit different chemical profiles?

In order to separate the effects of endosymbiont infection from herbivory on plant volatile profiles, we analyzed foliar volatile emissions in undamaged control plants and plants experimentally challenged with LB+ and LB− psyllids. Over 30 compounds, especially terpenoids, have been reported in the headspace of damaged potato plants, though most in trace amounts [25]. Therefore, we chose to assay ten compounds that are well-described and relatively abundant constituents of *S. tuberosum* headspace (β-caryophyllene, α-caryophyllene, β-sesquiphellandrene, [Z]-β-farnesene, β-cubebene, germacrene-D-4-ol, 3-hexen-1-ol, cyclohexene, caryophyllene oxide, and nonanal; [25], [26]. We employed a volatile collection system modified from that of Agelopoulos et al. [26] to isolate plant volatiles. We used plants that had been experimentally treated 6 d prior, and collections took place during the afternoon. Ambient conditions were 24°C and fluorescent lighting. We secured a 3 L glass chamber (guillotine chamber, Analytical Research Systems Inc., Gainesville, FL) over above-ground plant tissues and used a positive/negative pressure system to collect plant volatiles. We passed charcoal filtered, humidified air into glass chambers at a rate of 900 ml/min (positive pressure), and drew air from the chamber through a borosilicate volatile collection trap (VCT, 1.5 mm I.D.) packed with 20 mg of a crystalline polymer adsorbent material (HayeSep-Q) at a rate of 700 ml/min (negative pressure) for 1 h. This collection procedure was replicated for five plants from each treatment category, and system blanks were also collected from chambers prior to plant volatile collections.

We eluted VCTs with 200 µl of dichloromethane (CH_2_Cl_2_) and reduced extracts to ca. 10 µl under a stream of charcoal filtered nitrogen. We used a gas chromatograph coupled to a mass spectrometer (GC/MS) to analyze extracts. A 1 µl aliquot from each 10 µl extract was manually injected into a Hewlett Packard 6890 chromatograph (serial no. U.S.00031025) equipped with a Hewlett Packard 5973 mass selective flame ionization detector (FID). The GC column was a DB 5 Ultra Inert column (Agilent 122-5532UI) with internal dimensions of 0.25 µm×60 m. The GC was operated in splitless mode, and the carrier gas was He (48.67 kPa) at a flow rate of 19.3 ml/min. Starting temperature was 40°C for 2 min, then increasing by 10°C/min to 240°C and held for 2 min. Compound identifications were confirmed by matching retention times, Kovats indices, and electron mass spectra from eluted samples to those available in a commercial database (NIST 2005 Mass Spectral Library).

We used absolute concentrations (ion chromatograms) of compounds from GC traces to provide a quantitative basis for statistically analyzing differences in plant volatile emissions due to experimental treatments. We analyzed log-corrected peak areas from GC traces using one-way ANOVA, treating plant infection status as a fixed effect on the response variable of total ion chromatograms. We also performed a clustering analysis (Ward's method [27]) and a discriminant function analysis (linear method [28]) on peak areas to provide a statistical basis for grouping plant emissions by infection status in a multivariate context. We chose not to analyze relative (%) abundances of compounds since fewer peaks were found in air collections from control (undamaged) plants, and the relative proportions (%) of these peaks were thus excessively high.

### Does *B. cockerelli* select plants based on infection status?

We employed a modified release/recapture design to experimentally test psyllid plant selection behaviors on *S. tuberosum* plants with different infection statuses. Previous work in several closely related systems indicates that plant defense transcripts in solanaceous species are highly up-regulated 6 d following feeding by infected psyllids [29], and that host selection behaviors are equivalent among male and female psyllids and under lighted or dark conditions [16], [17]. Thus, we designed our experiments to reflect this time span, and the experiment was performed on a relevant photoperiod rather than under lighted or dark conditions only. After 6 d following experimental treatments, we placed one plant from each treatment group (undamaged control, LB+ treatment, and LB− treatment) into 60 cm×60 cm×60 cm mesh cages. We placed plants in a triangular array (ca. 50 cm/side), and we released 50 randomly collected LB− adult psyllids into each cage (*N* = 15) by placing an open vial containing psyllids in the center of the array. Plant positions were randomized in each cage. Cages were placed at a distance of 20 cm underneath fluorescent lights (SunBlaze T5HO-44, Lot no. 960300, Sunlight Supply, Inc., Vancouver, WA) in climate controlled rooms (24°C, 40% R.H., 16 h∶8 h L∶D).

We censused plants at 7, 8, 9, and 15 d following experimental challenges (1, 2, 3, and 8 d following release of psyllids) and recorded the number of psyllids on each plant. At 15 d, we terminated the experiment and censused the number of eggs oviposited by female psyllids on each plant. We then clipped and oven-dried (70°C for 24 h) the above-ground plant tissues and recorded dry mass (g) of tissues. Plant infection status was confirmed for the infected psyllid treatment group with PCR (primer pair OA2/OI2, as above) using below-ground plant material (stolons). We also recorded whether plants were flowering at the end of the experiment. We used repeat-measures ANOVA to analyze the effects of plant infection status (*n* = 3) on the response variable of mean number of psyllids/plant on each day (7, 8, 9, 10, and 15 d post-challenge), and number of eggs/plant at 15 d. We tested whether psyllid host selection was correlated with above-ground plant mass (g) using least-squares linear regression. We also assessed whether plant infection status (*n* = 3) affected the proportion of plants that were flowering at the end of the experiment using a chi-square test. We used the software JMP 9.0 (SAS Institute) to perform all statistical analyses, and all statistics incorporate a Type I error rate of α = 0.05. We visually confirmed homoscedasticity of variance using residual plots prior to hypothesis testing using parametric statistics.

### Does plant infection status affect *B. cockerelli* population growth?

We tested whether psyllid fecundity differed on infected and uninfected plants [15] by rearing psyllids on plants of different infection statuses. To assess psyllid population growth rates, we caged plants (*N* = 45; 15 plants in each treatment category) individually, as above, and released 3 virgin male and 3 virgin female psyllids onto each plant. All released psyllids were taken from uninfected colonies only (LB−). Fifteen days following the release of psyllids (21 d after experimental challenges), we terminated the experiment and assessed psyllid population growth by censusing the number of eggs and nymphs that were present on each plant. We again clipped and oven-dried (70°C for 24 h) the above-ground plant tissues and recorded dry mass (g) of tissues, and we recorded whether plants were flowering at the end of the experiment. We used ANOVA to test whether the mean number of eggs and the mean number of new psyllids (nymphs) differed by plant infection status (*n* = 3). We also tested whether psyllid reproductive performance was correlated with above-ground plant mass (g) or plant flowering status, using least-squares linear regression and one-way ANOVA, respectively.

## Results

### Do challenged plants exhibit different chemical profiles?

**Figure 1:**
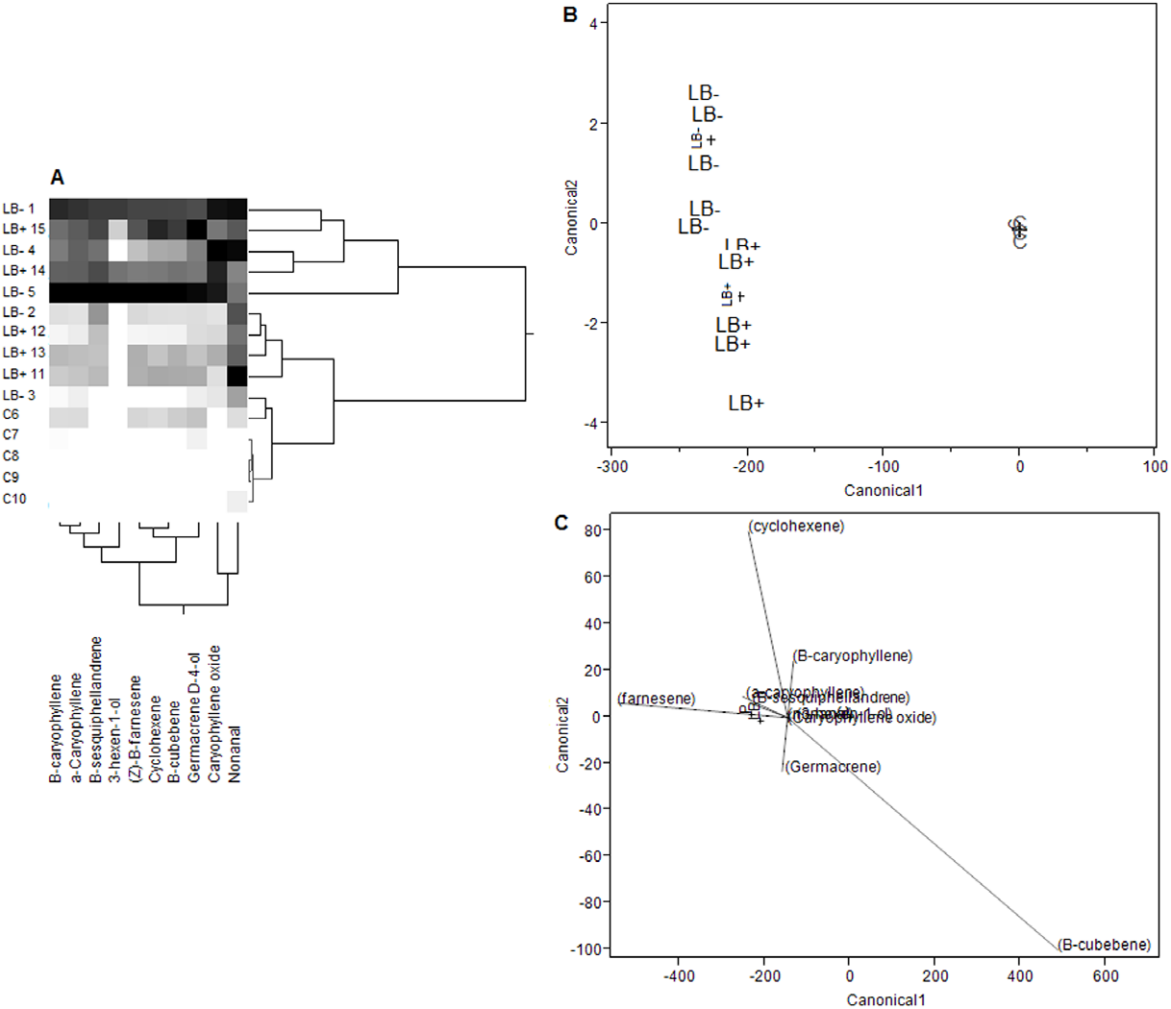
Analysis of volatile emissions from *S. tuberosum*. (A) Cluster analysis of the abundances (total ion chromatograms) of ten compounds in the headspace of undamaged control (C) and experimentally challenged (LB+, LB−) potato plants. Grayscale gradations show an increasing abundance of compounds from low to high (white to black, respectively). (B) A discriminant function analysis (DFA) plotting canonical scores of log corrected values of the ten compounds shown in cluster analysis. Individual plants are indicated by lettering (LB−, LB+, C) and crosses denote centroid values. (C) Biplot rays indicating the relative importance of each compound (shown by ray length) in the ordination. Note that the canonical variable scale differs from (B) to (C).

The abundance of volatile emissions, in terms of total ion chromatograms, were significantly lower for undamaged control plants than for either experimental treatment (LB+, LB−) for nine of the ten target compounds (Table 1). In univariate analyses, volatile emissions appeared similar for both experimental treatments (LB+, LB−). However, more distinctive groupings were identifiable in multivariate analyses. Clustering analysis indicated that, in general, plants challenged with LB− psyllids had a more severe response overall to herbivory, whereas plants challenged with LB+ psyllids also exhibited induction but to a lesser degree (Figure 1A). Discriminant function analysis (DFA) strongly supported the grouping of volatile emissions by experimental treatment (Wilks' λ = 60.026, df = 20, 6; *P*<0.0001), and volatile profiles of both LB+ and LB− treated plants were more similar to one another than to volatile profiles of control plants, yet distinct (Figure 1B, canonical coefficients shown in Table S1). The volatile compounds that appeared to play the strongest role in discriminating psyllid-exposed plants from undamaged controls were β-farnesene and β-cubebene; whereas cyclohexene, germacrene-D-4-ol, and β-caryophyllene appeared to be principally responsible for statistically separating LB+ from LB− plants (Figure 1C).

**Table 1:** ANOVA summary.

	Treatment			
Compound	Control	LB+	LB−	*F* a,b
β-caryophyllene	5.601 a	15.901 b	16.161 b	8.345**
α-caryophyllene	2.659 a	14.160 b	14.1495 a	17.408***
β-sesquiphellandrene	1.78^−15^ a	13.901 b	11.672 b	19.161***
[Z]-β-farnesene	2.769 a	14.494 b	12.181 b	6.577*
β-cubebene	2.771 a	14.463 b	12.012 b	6.594*
germacrene-D-4-ol	5.388 a	14.758 b	14.712 b	7.557**
3-hexen-1-ol	4.44^−16^	5.368	6.188	1.343
cyclohexene	2.770 a	14.717 b	12.273 b	6.741*
caryophyllene oxide	1.70^−15^ a	14.591 b	15.020 b	342.890****
nonanal	5.176 a	14.941 b	14.987 b	9.464**

a df = 2, 12;

b *P<0.05, ** *P*<0.01, ***P<0.001, ****P<0.0001.

Tabulated mean differences in total ion chromatograms for ten volatile compounds extracted from the headspace emissions of *S. tuberosum*. Values are log-transformed ion chromatograms (peak areas), and lettering shows Tukey's HSD test for each row.

### Does *B. cockerelli* select plants based on infection status?

**Figure 2:**
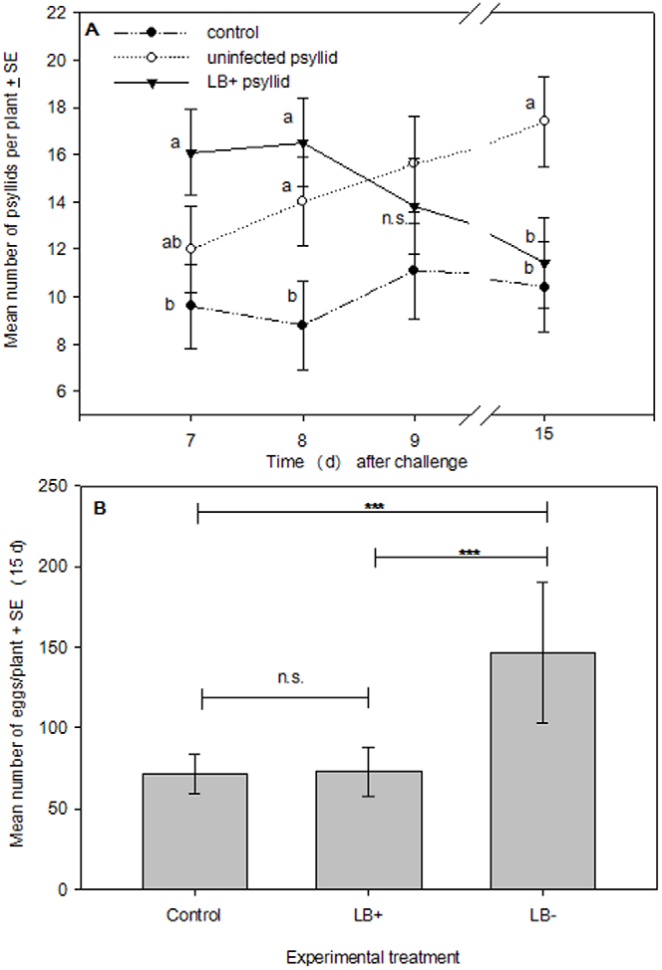
Psyllid settling and oviposition preferences. (A) Mean number of psyllids/plant/d^-1^ ± SE in a cage-release experiment (*N* = 45) testing the effects of *S. tuberosum* infection status on *B. cockerelli* settling behaviors, letters denote Tukey's HSD test; and (B) pairwise comparisons of mean number of eggs/plant ± SE at the end of the experiment (Bonferroni adjusted Type I error rate: α = 0.016; *** *P*<0.001).

Our repeat-measures ANOVA model indicated a significant day x treatment interaction on psyllid host selection behavior (*F*_8, 196_ = 2.151; *P* = 0.032) as well as a strong effect from experimental treatments alone (*F*_2, 196_ = 13.835; *P*<0.0001); however day alone did not have a significant effect on psyllid host selection behavior (*F*_4, 196_ = 1.268; *P* = 0.283). As a result, we directly analyzed day x treatment interactions using Tukey's HSD test at each census point. At 7 d following experimental challenges, plants that were experimentally exposed to infected psyllids attracted significantly more psyllids than undamaged control plants on average, and at 8 d following experimental challenges both treatment groups (psyllid only and LB+ psyllid) had significantly more psyllids on plants on average than untreated controls. At 9 d following experimental challenges, there were no significant differences in the mean number of psyllids/plant due to experimental treatments, but at 15 d following experimental challenges plants that were experimentally exposed to uninfected psyllids had higher mean numbers of psyllids than either control plants or plants experimentally exposed to infected psyllids (Figure 2A).

At 15 d following experimental challenges, psyllids had oviposited approximately 51.1% and 50.3% more eggs on plants exposed to uninfected psyllids than undamaged control plants or plants exposed to infected psyllids, respectively. This difference trended towards significance in our ANOVA model (*F*_2, 42_ = 2.433, *P* = 0.100), but there was considerable variance in the number of eggs per plant. However, we employed Students *t*-tests to make all pairwise comparisons (*n* = 3) using an adjusted Type I error rate (Bonferroni's correction; α = 0.016), and found that significantly fewer eggs were laid on control plants (*t*_14_ = −6.204; *P*<0.001) and plants exposed to infected psyllids (*t*_14_ = −4.882; *P*<0.001; Figure 2B) than plants that had been exposed to psyllids only.

We did not find any evidence to suggest that above-ground plant biomass was correlated with psyllid host selection at 7 d (*F*_1, 43_ = 2.629, *r* = 0.057, *P* = 0.112), 8 d (*F*_1, 43_ = 2.194, *r* = 0.048, *P* = 0.145), 9 d (*F*_1, 43_ = 2.291, *r* = 0.050, *P* = 0.137), or 15 d (*F*_1, 43_ = 1.053, *r* = 0.023, *P* = 0.310) following experimental challenges. Similarly, we did not find any evidence that oviposition (eggs/plant) by psyllids was correlated with above-ground plant biomass (*F*_1, 43_ = 0.039, *r* = 0.000, *P* = 0.843). Interestingly, our experimental treatments appeared to have a modest effect on plant sexual reproduction: we found that 26.7% of control plants were flowering by the end of the study, compared with 40% of plants treated with infected psyllids and 66.7% of plants treated with uninfected psyllids (χ^2^ = 5.143; df = 2; *P* = 0.076).

### Does plant infection status affect *B. cockerelli* population growth?

Psyllids reproduced prolifically over the 15 d period of the experiment, and we counted a total of 7,347 nymphs and 8,553 eggs on 45 plants. However, mean psyllid population growth rates did not vary by experimental treatments (eggs/plant: *F*_2, 42_ = 0.312; *P* = 0.733; nymphs/plant: *F*_2, 42_ = 0.527; *P* = 0.594). On average, we counted 189.8±25.4 eggs and 157.1±18.31 nymphs on control plants; 203.0±19.94 eggs and 181.1±21.13 nymphs on uninfected plants; and 177.3±23.13 eggs and 151.6±25.95 nymphs on infected plants.

**Figure 3:**
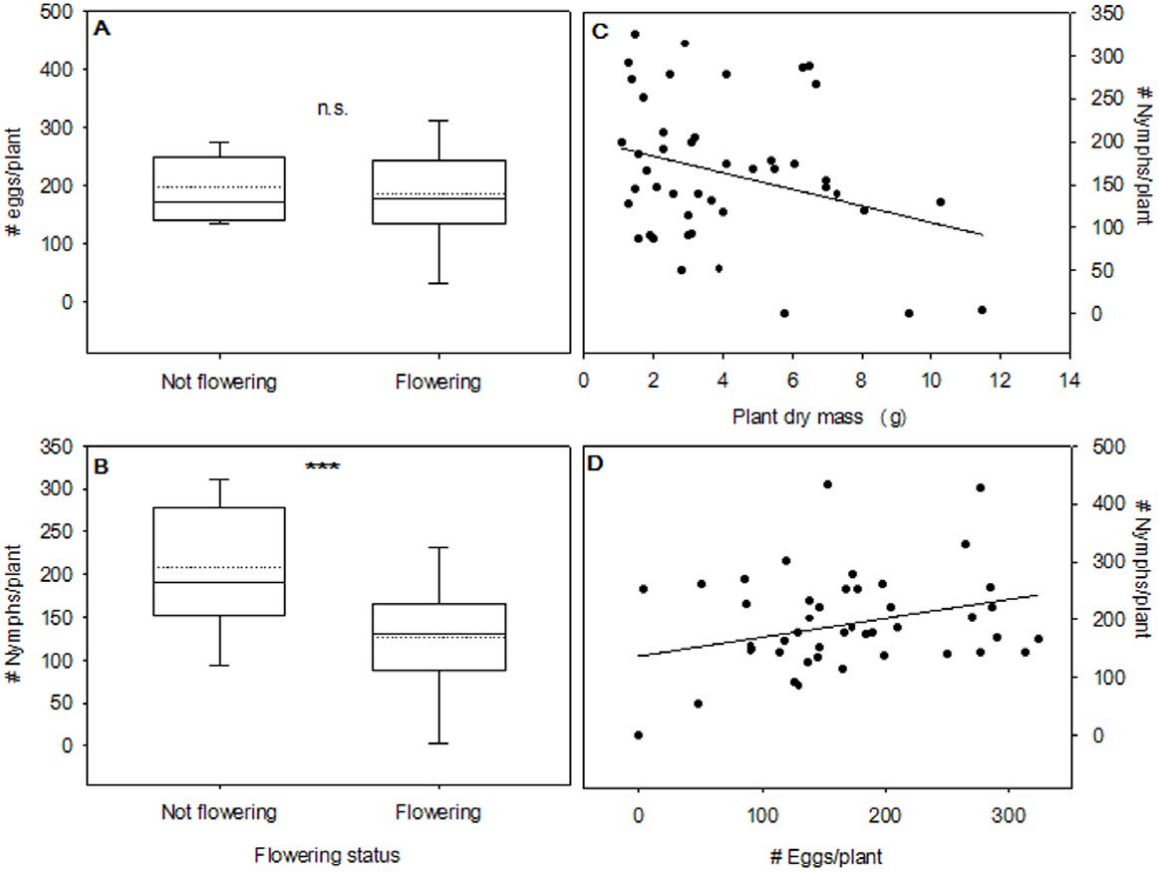
Psyllid rearing experiment. (A) Box-and-whiskers plot denoting the distribution of psyllid eggs/plant by flowering status; (B) Box-and-whiskers plot representing the distribution of psyllid nymphs/plant by flowering status (***P<0.001); (C) the correlation between plant size and psyllid nymphs/plant (*N* = 45, regression equation: [nymphs/plant] = 202.304−9.625 [Plant dry mass (g) ]); and (D) the correlation between psyllid oviposition (# eggs/plant) and nymph production (#nymphs/plant; *N* = 45, regression equation: [nymphs/plant] = 107.677+0.292 [eggs/plant]). The dotted lines in (A) and (B) show the mean of each distribution.

Interestingly, plant sexual reproduction (flowering) was correlated with psyllid reproductive performance. There was no significant relationship between flowering and the mean number of eggs per plant (*F*_1, 43_ = 0.154; *P* = 0.696; Figure 3A), however; psyllids produced on average 38.67% fewer nymphs on plants that were flowering at the end of the study (flowering: 127.52±14.64 nymphs, not flowering: 207.95±16.37; *F*_1, 43_ = 12.409; *P* = 0.0007; Figure 3B). In addition, plant size had a weak negative correlation with psyllid reproduction: larger plants had fewer nymphs (*F*_1, 43_ = 4.282; *P* = 0.044; *r* = −0.301; Figure 3C), but the number of eggs/plant was not correlated with plant size (*F*_1, 43_ = 0.779; *P* = 0.382). Larger plants were also more likely to be flowering at the end of the experiment (χ^2^ = 6.59; df = 1; *P* = 0.010). Not surprisingly, we also found that the number of eggs per plant was modestly correlated with the number of nymphs per plant (*F*_1, 43_ = 4.530; *P* = 0.039; *r* = 0.308; Figure 3D).

## Discussion

Consistent with other recent research, our volatile collection experiment supported the hypothesis that infection with the endosymbiotic “*Ca*. L. solanacearum” can alter plant (*S. tuberosum*) volatile emissions [15]–[18], [29], [30]. The major volatile components extracted and identified from *S. tuberosum* headspace with our collection procedure were concordant with those identified by other researchers from *S. tuberosum* headspace (e.g., [25], [26], [31]). The majority of potato headspace volatiles were sesquiterpenes such as β-caryophyllene, β-sesquiphellandrene, and [Z]-β-farnesene; though green leaf volatiles such as 3-hexen-1-ol were also detected. Our results suggest that, in terms of abundance, the effects of herbivory alone differ from the effects of herbivory plus endosymbiont infection (Fig. 1B). However, plants that were exposed to herbivory were more similar to one another in terms of compound abundance than to undamaged control plants, regardless of herbivore infection status. Most previous studies directly inoculated plants or grafted infected tissues onto uninfected plants, whereas our studies utilized actual herbivores infected with a pathogenic endosymbiont (“*Ca*. L. solanacearum”) to transmit infection to plants. This approach may provide a more realistic context for the comparison of plant volatile emissions between symbiont-infected and undamaged individuals, as conditions in the field probably reflect a mosaic pattern of herbivore-damaged but uninfected plants, damaged and infected plants, undamaged plants, and possibly defensively induced plants with no prior exposure to herbivory [32], [33].

Our psyllid-release experiment was consistent with the hypothesis that plant infection impacts the settling behavior of psyllids. Initially, more psyllids on average settled on plants previously exposed to endosymbiont-infected psyllids. However, psyllids gradually defected to plants that had only been previously exposed to uninfected psyllids (Fig. 2A). This finding has potential implications for the epidemiology of “*Ca.* L. solanacearum”: we speculate that psyllids are likely to preferentially settle on plants infected with “*Ca.* L. solanacearum”, become infected with the symbiont, and subsequently transmit the symbiont to new host plants by defecting to uninfected plants. Currently, little is known regarding at which point during their lifecycle psyllids acquire “*Ca.* L. solanacearum”, although it has been demonstrated that feeding for as little as 8 h on infected *S. tuberosum* plants is sufficient to acquire the endosymbiont [35]. In addition, there appears to be a latency period lasting up to two weeks following inoculation, during which visible symptoms of “*Ca.* L. solanacearum” [35] are not expressed. Accordingly, it seems plausible that the primary vectors of “*Ca.* L. solanacearum” are adult psyllids, and Buchman et al. [24] demonstrated that adults were more effective vectors of “*Ca.* L. solanacearum” than nymphs. Whether the symbiont is transmissible during the latency period is currently unknown. Interestingly, the latency period matched our experimental results for psyllid defection: by 15 d, a majority of psyllids had defected from LB+ to LB− plants.

A similar result was demonstrated by Mauck et al. [15], Mann et al. [16], and McMenemy et al. [17], indicating that an initial preference by herbivores for endosymbiont-infected plants followed by defection may be a common behavioral pattern in herbivore-plant-endosymbiont interactions. These studies collectively demonstrate that endosymbionts can indirectly affect herbivore behaviors by altering plant chemical signals. Other recent studies have also demonstrated that endosymbionts may have direct effects on herbivore behaviors. For example, Ingwell et al. [36] showed that herbivores artificially inoculated with viral endosymbionts during *in vitro* feeding assays preferred uninfected plants, whereas uninfected individuals preferred infected plants. Future work in herbivore-endosymbiont-plant research should attempt to address both the direct and indirect effects of endosymbionts on herbivore behaviors. It will be important for researchers to determine, across multiple ecological systems, whether the acquisition and transmission of herbivore-associated endosymbionts is generally consistent with a ‘deceptive host phenotype’ hypothesis [15], a ‘vector manipulation’ hypothesis [36], or whether elements of both direct and indirect effects mediate symbiont acquisition and transmission. One shortcoming of our experimental design should be noted: as we only controlled for the initial exposure of plants of infected and uninfected psyllids, the possibility remains that the change over time we observed in plant preference by psyllids was due to the transmission of “*Ca.* L. solanacearum” to LB− plants, thereby increasing their attractiveness. However, if this were the case, we might also expect a corresponding increase in the attractiveness of undamaged control plants, which was not observed.

Unfortunately, the design of our studies does not allow us to definitively state that olfaction was the mechanism for psyllid orientation to infected plants: our experiment incorporated components of olfaction, but odor sources were not tested in isolation. However, our findings do appear to be correlated with plant volatile emissions, and as above we found a substantial difference in emissions between plants exposed to “*Ca.* L. solanacearum” and control plants. Alternatively, some researchers have demonstrated that visual and gustatory cues, rather than olfactory cues, can mediate herbivore discrimination among suitable hosts or infected and uninfected plants [30], [34]. Some yellowing of plants was observed in our experiments, and although relatively minor, could create a potentially important visual cue. Based on our findings, future work on this system should seek to determine whether the close-range orientation response and resulting host plant preference of *B. cockerelli* are explainable by visual cues, olfactory cues, gustatory cues, or some combination of these stimuli.

In contrast to their initial tendency to settle on infected (LB+) plants, mean psyllid oviposition (number of eggs layed) was higher on plants that were exposed to uninfected psyllids (Fig. 2B). In a recent study, Nachappa et al. [22] found that the fecundity of female psyllids was reduced by infection with “*Ca.* L. solanacearum”, which could partially explain the reduced rates of oviposition we observed on LB+ plants if adults acquired the endosymbiont while feeding, and then subsequently oviposited on infected plants. We also found in a post hoc analysis that a greater proportion of plants experimentally exposed to psyllids (i.e., LB+ and LB− treatments) were flowering by the end of the psyllid release experiment, suggesting a potential correlation between exposure to herbivory and flowering phenology in *S. tuberosum*.

Contrary to our expectations, our psyllid rearing experiment on LB+, LB−, and undamaged control plants did not reveal a substantial effect of plant exposure to endosymbionts on psyllid population growth. In other works, plants that became infected with bacterial or viral endosymbionts significantly reduced herbivore population growth [15], increased the development time of herbivores [17], and decreased the concentration of macronutrients in plant tissues [16]. However, the opposite trend has also been demonstrated: Belliure et al. [37] found that thrips populations benefitted in terms of survival and development time when plants were inoculated with wilt virus. One possible discrepancy between our experiment and previous work is that *B. cockerelli* does not reproduce by parthenogenesis. Alternatively, any mortality of parent psyllids could introduce substantial variance to subsequent estimates of population performance, since relatively few psyllids were placed with each plant. Despite this potential shortcoming of the experimental design, psyllid reproduction was prolific, suggesting that psyllids did not encounter any apparent difficulty initiating mating and oviposition behaviors.

Interestingly, both flowering phenology and plant size were found to affect estimates of psyllid population growth. Plants that were flowering at the end of the experiment had significantly fewer nymphs than plants which were not flowering at the end of the experiment (Fig. 3B). Similarly, an increase in plant biomass corresponded with a decrease in the number of nymphs found on plants (Fig. 3C), and plant biomass was positively correlated with flowering status. When coupled with the finding that exposure to psyllids appeared to prompt flowering, novel questions emerge: (1) does exposure to herbivores facilitate flower set in *S. tuberosum*?; and (2) does flowering reliably correlate with herbivore performance? Although we did not undertake to answer these questions, our results provide anecdotal evidence that there may be important ecological feedbacks between herbivory, flowering phenology, and herbivore performance [38]–[40]. To our knowledge, no studies have specifically addressed whether herbivory can prompt flower set.

In summary, our results generally support a ‘deceptive host phenotype’ hypothesis [15]: plant infection with herbivore-associated endosymbionts impacted plant volatile emissions, herbivore settling behaviors, and oviposition preferences. We found that the effect of herbivory on plant volatile emissions differed from the effects of herbivory plus endosymbiont infection, and that psyllids initially settled on infected plants but gradually defected to plants only previously exposed to uninfected psyllids. Psyllids also preferentially oviposited on the plants they defected to. However, we did not find any evidence to suggest that psyllid population dynamics were affected by exposing plants to psyllids colonized by endosymbionts. Our findings, coupled with other recent studies, have implications for the epidemiology of plant-pathogenic endosymbiotic associations of phloeophagous herbivores: in multiple systems, uninfected herbivores appear to initially prefer infected plants, and then subsequently migrate to uninfected plants, potentially transmitting the pathogenic endosymbiont to new hosts. Further work addressing the physiological mechanisms responsible for psyllid attraction to infected hosts, host legacy effects on psyllid reproduction, and ecological interactions between herbivory and flowering phenology would all strengthen our understanding of the ecology of this system.

## Supporting Information

Table S1 Standardized scoring coefficients. The importance of each compound in separating plant headspaces by experimental treatments along discriminant function (DF) axes 1 and 2. Values are dependent variable canonical coefficients, and absolute values rank the weight of each compound in the ordination.
(TIF)
