# Supplementary figures and images for: Experimental Infection of Plants with an Herbivore-Associated Bacterial Endosymbiont Influences Herbivore Host Selection Behavior
Source: PLoS One. 2012 Nov 14;7(11):e49330. doi: 10.1371/journal.pone.0049330 (PMC3498155; doi:10.1371/journal.pone.0049330)

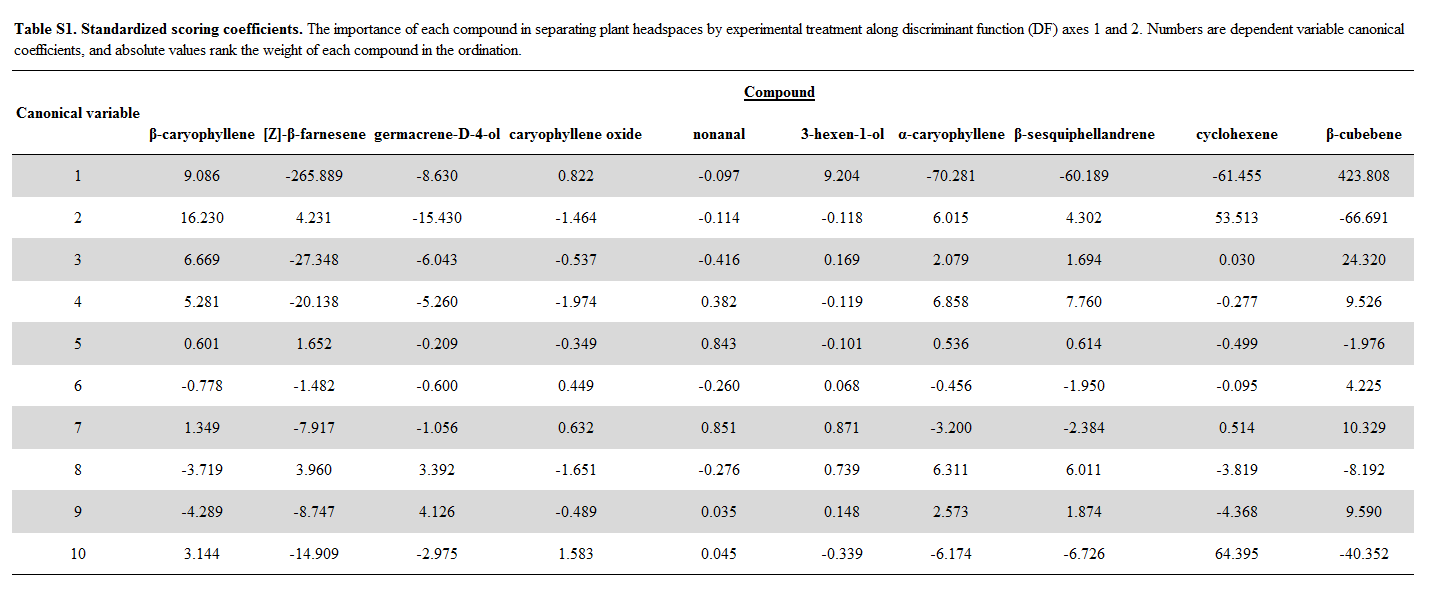

Supplement: Table S1 — Standardized scoring coefficients. The importance of each compound in separating plant headspaces by experimental treatments along discriminant function (DF) axes 1 and 2. Values are dependent variable canonical coefficients, and absolute values rank the weight of each compound in the ordination. (TIF) [file pone.0049330.s001.tif]
